# 3D single shot lensless incoherent optical imaging using coded phase aperture system with point response of scattered airy beams

**DOI:** 10.1038/s41598-023-30183-0

**Published:** 2023-02-21

**Authors:** Ravi Kumar, Vijayakumar Anand, Joseph Rosen

**Affiliations:** 1 School of Electrical and Computer Engineering, Ben-Gurion University of the Negev, P.O. Box 653, 8410501 Beer-Sheva, Israel; 2 Department of Physics, SRM University-AP, Amaravati, Andhra Pradesh 522502 India; 3 Institute of Physics, University of Tartu, W. Ostwaldi 1, 50411 Tartu, Estonia; 4 Optical Sciences Center, Swinburne University of Technology, Hawthorn, Melbourne, VIC 3122 Australia; 5 Stellenbosch Institute for Advanced Study (STIAS), Wallenberg Research Centre at Stellenbosch University, Stellenbosch, 7600 South Africa

**Keywords:** Imaging and sensing, Optics and photonics

## Abstract

Interferenceless coded aperture correlation holography (I-COACH) techniques have revolutionized the field of incoherent imaging, offering multidimensional imaging capabilities with a high temporal resolution in a simple optical configuration and at a low cost. The I-COACH method uses phase modulators (PMs) between the object and the image sensor, which encode the 3D location information of a point into a unique spatial intensity distribution. The system usually requires a one-time calibration procedure in which the point spread functions (PSFs) at different depths and/or wavelengths are recorded. When an object is recorded under identical conditions as the PSF, the multidimensional image of the object is reconstructed by processing the object intensity with the PSFs. In the previous versions of I-COACH, the PM mapped every object point to a scattered intensity distribution or random dot array pattern. The scattered intensity distribution results in a low SNR compared to a direct imaging system due to optical power dilution. Due to the limited focal depth, the dot pattern reduces the imaging resolution beyond the depth of focus if further multiplexing of phase masks is not performed. In this study, I-COACH has been realized using a PM that maps every object point into a sparse random array of Airy beams. Airy beams during propagation exhibit a relatively high focal depth with sharp intensity maxima that shift laterally following a curved path in 3D space. Therefore, sparse, randomly distributed diverse Airy beams exhibit random shifts with respect to one another during propagation, generating unique intensity distributions at different distances while retaining optical power concentrations in small areas on the detector. The phase-only mask displayed on the modulator was designed by random phase multiplexing of Airy beam generators. The simulation and experimental results obtained for the proposed method are significantly better in SNR than in the previous versions of I-COACH.

## Introduction

Incoherent digital holography (IDH) techniques have been rapidly evolving due to the demands of fluorescence microscopy and astronomical and biomedical imaging^1–4^. The fundamental task of IDH technologies is to encode three-dimensional (3D) information of the object using between one to four intensity patterns and reconstruct the 3D image numerically. Conventionally, the encoding procedure involves recording a self-interference hologram between two differently modulated object waves^1^. This self-interference hologram numerically propagates to reconstruct the object’s image. The number of recordings varies depending on whether the optical configuration is inline or off-axis. An inline configuration requires at least three camera shots to remove the twin image and bias terms. An off-axis configuration requires only a single shot but has a limited field of view.

In 2007, Fresnel incoherent correlation holography (FINCH) was proposed, which is still one of the simplest, most robust, and compact among IDHs to date^5^. Furthermore, FINCH has also exhibited a resolution exceeding the resolution limit by breaking the Lagrange invariant conditions^1,6^. However, the FINCH systems require a dynamic electro-optical device to create phase-shifted phase masks and record multiple camera shots. Because of the two-beam interference, these systems need a vibration isolation setup, several phase modulators (PMs), and polarizers. Many techniques have been developed to achieve a single shot in FINCH by polarization multiplexing^7^, spatial multiplexing^8,9^, and avoiding using dynamic devices^10–12^, but the performances were not as good as the optimal FINCH^13^. In this line of research, an incoherent digital holography technique called coded aperture correlation holography (COACH) was developed^14^. The main difference between FINCH and COACH is that in the case of FINCH, the light from an object point is split into two beams, each of which is modulated by a different quadratic phase mask. In COACH, one object beam is modulated by a quasi-random phase mask, and the other experiences a constant value phase mask. In FINCH, the complex hologram obtained after phase shifting and the resulting hologram numerically back propagates, reconstructing the 3D image. In COACH, in the first step, point spread holograms (PSHs) are recorded for different depths of the point, followed by recording an object hologram. The object’s image is reconstructed by cross-correlating the PSHs with the object hologram. The imaging characteristics of FINCH and COACH are quite different. FINCH exhibits a lateral resolution of 1.5 times that of incoherent imagers with the same NA and low axial resolution and has a low reconstruction noise, but COACH has lateral and axial resolutions similar to those of a regular lens-based imaging system and a higher reconstruction noise^15^.

In 2017, during COACH research, it was identified that the two-beam interference in COACH is redundant. Recall that the fundamental task of IDH is to encode 3D information uniquely. In COACH, the scattered intensity distribution from the quasi-random phase mask is unique for every axial location of the input point, even without interference. This observation led to the development of interferenceless COACH (I-COACH)^16^ and, later, a simplified version of lensless I-COACH (LI-COACH)^17^. In both I-COACH and LI-COACH, the phase mask was extensively engineered to achieve a high SNR with controlled scattering. A statistical averaging method was applied in the case of both I-COACH and LI-COACH to further improve the SNR. Later, a novel reconstruction method called non-linear reconstruction (NLR) was developed to improve the reconstruction results in I-COACH and LI-COACH^18^. A low-cost version of LI-COACH was demonstrated using a mask containing a quasi-random array of pinholes to image objects in four dimensions^19^. In this line of research, many imaging techniques, such as DiffuserCam^20^ and scatter-plate microscopes^21–23^, were developed. Some of these methods make use of off-the-shelf diffusers, and thus the aperture masks are not optimized for various applications, as is the case in the present study. In general, off-the-shelf diffusers are lossy as they absorb some of the light, and their transmission function cannot be engineered according to the desired application. Several studies have shown a low field of view resulting from the low memory effect with off-the-shelf diffusers^24^.

One of the main challenges in using scattering masks is the low SNR compared to a direct imaging system, which makes them unsuitable for light-sensitive applications. This is true especially when recording the point spread functions (PSFs), as the light throughput through a pinhole is significantly low, resulting in the signal reaching the noise level of the detector. Unfortunately, improving the light throughput by increasing the size of the pinhole reduces the imaging resolution. Recalling in I-COACH, the lateral resolution limit is approximately given by the average speckle size, which is governed by the NA of the system. However, there is a secondary resolution limit in I-COACH, which is set by the size of the pinhole used for recording the PSF. Therefore, there is a trade-off between the SNR and lateral resolution. One direction that was pursued to improve SNR and relax the above trade-off was to replace the scattered intensity distribution with a few randomly located dots^25^ and ring patterns^26^. The main problem with this approach was the need for intensive multiplexing of phase masks to image the 3D scene at different depths with the maximum diffraction-limited resolution^27^. Recently, the fundamental building block of speckles was engineered using Bessel beams, and the possibility of tuning the axial resolution independent of lateral resolution was demonstrated^28^.

The above studies lead to an interesting question: What is an optimal phase mask for LI-COACH? For optimal performance, the phase mask of LI-COACH must scatter light in a controlled manner. In other words, the system’s PSF should be focused on a limited area to deliver maximum intensity. On the other hand, the PSF’s autocorrelation should be as sharp as possible for different depths of the input point and with a relatively low cross-correlation value between PSFs of different depths. Intuitively, the above requirements can be achieved if the phase mask satisfies the following conditions: (a) The total area of the PSF is relatively small for any depth of the input point in a given range. (b) The structure of the PSF along the input point’s depth is changed randomly. A promising candidate to satisfy the above requirements is the Airy beam^29^. In this study, a sparse random array of Airy beams was investigated for 3D incoherent imaging. Airy beams are peculiar, following a curved path of propagation in free space^29–31^. Airy beams are non-diffractive and have self-healing properties, which make them excellent candidates for a wide range of imaging applications^32^. Most of the studies of Airy beams have used mostly coherent light sources. In a recent study, it was established that incoherence does not affect the special characteristics of Airy beams^33^. In general, an Airy beam can be directly used for 3D imaging with NLR. However, due to the long focal depth of the Airy beam, the axial resolution is low, which makes it unsuitable for 3D imaging. In this study, the axial resolution is improved by using multiple Airy beams with different characteristics and propagation directions. Therefore, an axial shift in the object location will cause a lateral shift of the intensity spot created by each Airy beam on the sensor plane, and a complete change in the system intensity response. This behavior results in the generation of a unique intensity distribution for every 3D location of the object. The high focal depth of Airy beams and the randomness associated with the uncorrelated shifts and tilts of the Airy beams with respect to one another offer the possibility to tune the axial resolution independently of the lateral resolution.

The manuscript consists of five sections. The next section describes the theoretical analysis and the methodology. In the third section, the design procedure and simulation results are presented. The experimental procedure and the results are described in the fourth section, followed by the conclusion in the final section.

## Methodology

**Figure 1:**
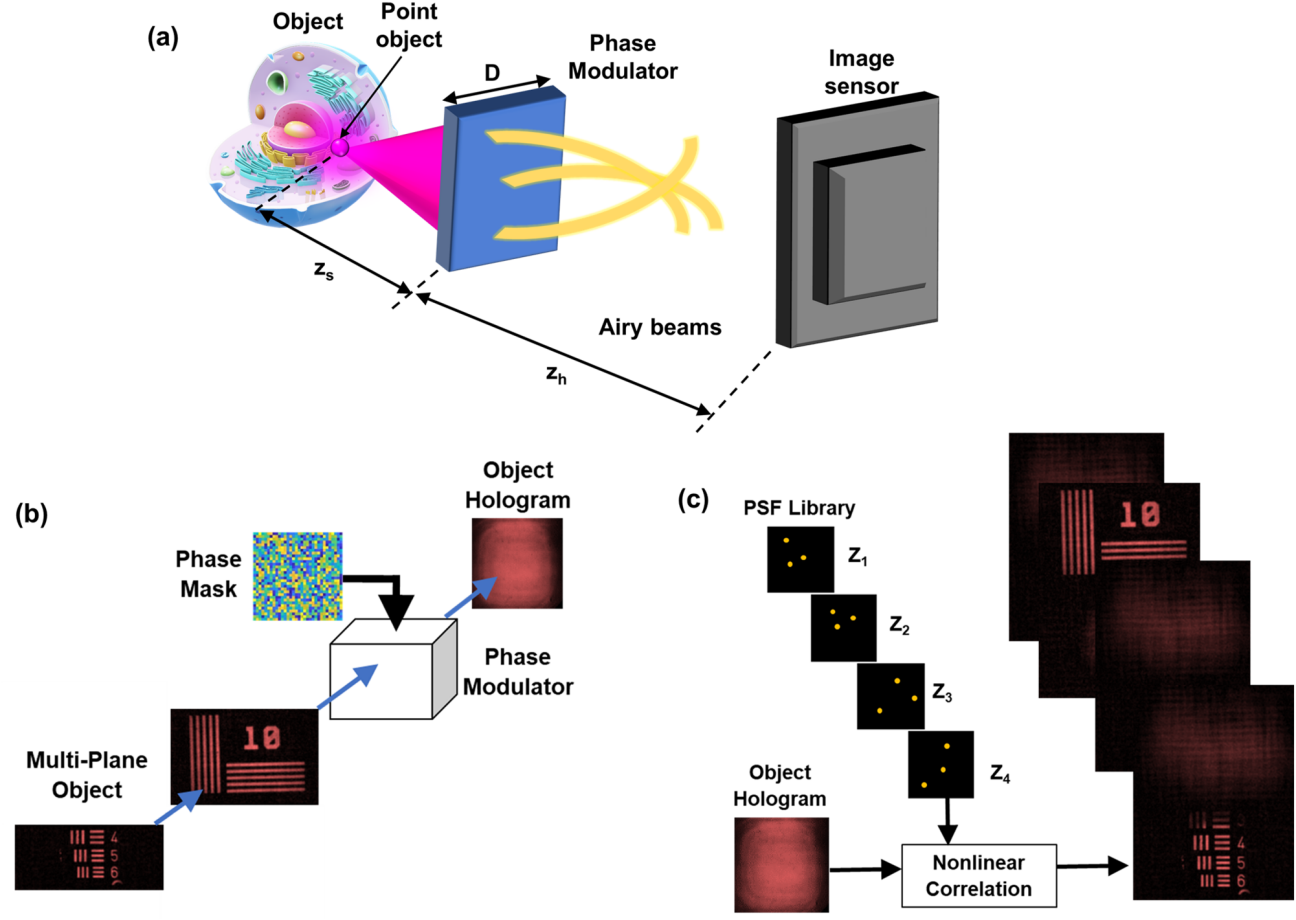
(**a**) Optical scheme of the imaging concept. Light from an object point is converted into a sparse random array of Airy beams. (**b**) Recording procedure: light from a multiplane object is incident on a phase modulator, and the object hologram is recorded. (**c**) Reconstruction procedure: the object hologram is processed with the PSF library reconstructing depth-specific information. The images in (**c**) are generated using MATLAB (version R2020b)^36^.

The conceptual scheme of the proposed 3D imaging method is shown in Fig. 1. The imaging concept, recording and reconstruction procedures are shown in Fig. 1a–c, respectively. Light from an object point located at $\left(\bar{r}_{s},{z}_{s}\right)=\left({x}_{s},{y}_{s},{z}_{s}\right)$ with an amplitude of $\sqrt{{I}_{s}}$ is incident on a PM located at a distance of *z*_*s*_. The complex amplitude of the PM is given by $Rect\left(\frac{x}{D},\frac{y}{D}\right)\mathrm{exp}\left(j\Phi \right).$ Hence, the complex amplitude beyond the PM is given by $\sqrt{{I}_{s}}{C}_{1}L\left(\frac{\bar{r}_{s}}{{z}_{s}}\right)Q\left(\frac{1}{{z}_{s}}\right)Rect\left(\frac{x}{D},\frac{y}{D}\right)\mathrm{exp}\left(j\Phi \right)$, where *L* and *Q* are the linear and quadratic phase functions given as $L\left(\frac{\overline{s}}{z }\right)=\mathit{ex}p\left[i2\pi {\left(\lambda z\right)}^{-1}\left({s}_{x}x+{s}_{y}y\right)\right]$ and $Q(b)=\mathit{exp}\left[i\pi b{\lambda }^{-1}\left({x}^{2}+{y}^{2}\right)\right]$, respectively, and *C*_1_ is a complex constant. The PM generates multiple Airy beams with different propagation directions, and the intensity distribution is recorded by an image sensor located at a distance of *z*_*h*_ from the PM. The PSF can be expressed as

1 $${I}_{PSF}\left(\bar{r}_{0};{\overline{r} }_{s},{z}_{s}\right)=\left|\sqrt{{I}_{s}}{C}_{1}L\left(\frac{\bar{r}_{s}}{{z}_{s}}\right)\right.Q\left(\frac{1}{{z}_{s}}\right)Rect\left(\frac{x}{D},\frac{y}{D}\right)\mathrm{exp}\left(j\Phi \right){\left.*Q\left(\frac{1}{{z}_{h}}\right)\right|}^{2},$$

where the symbol ‘ ∗ ’ denotes a two-dimensional convolution and $\bar{r}_{0}=(u,v)$ is the transverse location vector on the sensor plane. The above intensity distribution can be expressed as ${I}_{PSF}\left(\bar{r}_{0};{\overline{r} }_{s},{z}_{s}\right)={\left|\sum_{i=1}^{N}{Ai}_{i}\left(\frac{{x}_{i}}{{a}_{i}},\frac{{y}_{i}}{{a}_{i}}\right)\right|}^{2}$, where *Ai* is the Airy function, *N* is the number of Airy beams, *x*_*i*_ and *y*_*i*_ are the transformed coordinates given as ${x}_{i}={x}_{0}cos{\theta }_{i}+{y}_{0}sin{\theta }_{i}$, ${y}_{i}={y}_{0}cos{\theta }_{i}-{x}_{0}sin{\theta }_{i}$, ${\theta }_{i}\in \left[\mathrm{0,2}\pi \right]$ and *a*_*i*_ is the scaling factor of the truncated Airy beam^34,35^.

As the distances used in the optical configuration are large, a paraxial approximation can be used to simplify the theoretical calculations. Under the paraxial approximation, the intensity distribution for an off-axis point object results in a shifted version of the intensity distribution for a point object located on the optical axis $(\bar{r}_{s}=0)$, i.e., a spherical wavefront approximated by a quadratic wavefront. The amount of the shift is $\bar{r}_{s}{z}_{h}/{z}_{s}$, where ${z}_{h}/{z}_{s}$ is the magnification *M*_*T*_, and the following relation is satisfied,

2 $${{I}_{PSF}\left(\bar{r}_{0};{\overline{r} }_{s},{z}_{s}\right)=I}_{PSF}\left(\bar{r}_{0}-\frac{{z}_{h}}{{z}_{s}}\bar{r}_{s};0,{z}_{s}\right).$$

A 2D object can be expressed as a collection of Kronecker Delta-like functions given as

3 $$o\left(\bar{r}_{s}\right)=\sum \limits_{k}^{M}{g}_{k}\delta \left({\bar{r}} - {\bar{r}}_{s,k}\right),$$

where *M* is the number of object points and ${g}_{k}{^{\prime}}s$ are constants. In this study, spatially incoherent illumination is used, so the light emitted from one object point does not interfere with light from another point, but there is only an intensity accumulation in the sensor plane. Therefore, the object intensity distribution can be given as

4 $${I}_{O}\left(\bar{r}_{0};{z}_{s}\right)=\sum \limits_{k}^{M}{g}_{k}{I}_{PSF}\left(\bar{r}_{0}-\frac{{z}_{h}}{{z}_{s}}\bar{r}_{s,k};0,{z}_{s}\right).$$

The image of the object can be reconstructed by correlating ${I}_{O}\left(\bar{r}_{0};{z}_{s}\right)$ with ${I}_{PSF}\left(\bar{r}_{0};{z}_{s}\right)$ as follows,

5 $$ \begin{aligned} P\left(\bar{r}_{R}\right) & =\iint {I}_{O}\left(\bar{r}_{0};{z}_{s}\right){I}_{PSF}^{*}\left(\bar{r}_{0}-\bar{r}_{R};{z}_{s}\right)d\bar{r}_{0} \\ & =\iint \sum \limits_{k}{g}_{k}{I}_{PSF}\left(\bar{r}_{0}-\frac{{z}_{h}}{{z}_{s}}\bar{r}_{s,j};{z}_{s}\right){I}_{PSF}^{*}\left(\bar{r}_{0}-\bar{r}_{R};{z}_{s}\right)d\bar{r}_{0} \\ & = \sum \limits_{k}{g}_{k}\Lambda \left(\bar{r}_{R}-\frac{{z}_{h}}{{z}_{s}}\bar{r}_{s,j}\right)\approx o\left(\frac{\bar{r}_{s}}{{M}_{T}}\right), \end{aligned}$$

If Λ is a *δ*-like function, i.e., ~ 1 at (0,0) and ~ 0 elsewhere, then the object’s image is properly sampled. However, in a regular correlation, commonly known as the matched filter correlation, there is substantial background noise, as both functions *I*_*O*_ and *I*_*PSF*_ are positive functions, and the width of the autocorrelation function is at least twice that of the average size of the spot recorded in the sensor plane. In this study, NLR is applied, which reduces the background noise and sharpens the autocorrelation function close to that of the diffraction-limited spot. The NLR is given as ${I}_{R}=\left|{\mathcal{F}}^{-1}\left\{{\left|{\widetilde{I}}_{PSF}\right|}^{\alpha }\mathrm{exp}\left[j\cdot \mathrm{arg}\left({\widetilde{I}}_{PSF}\right)\right]{\left|{\widetilde{I}}_{O}\right|}^{\beta }\mathrm{exp}\left[-j\cdot \mathrm{arg}\left({\widetilde{I}}_{O}\right)\right]\right\}\right|$, where *α* and *β* are real numbers searched until the lowest reconstruction noise quantified by the entropy is obtained, arg(·) refers to the phase, and $\widetilde{I}$ is the Fourier transform of *I*. It has been recently observed that NLR applied to different types of deterministic optical fields generated a reconstruction spot close to the diffraction-limited spot^37^.

## Design and simulations

**Figure 2:**
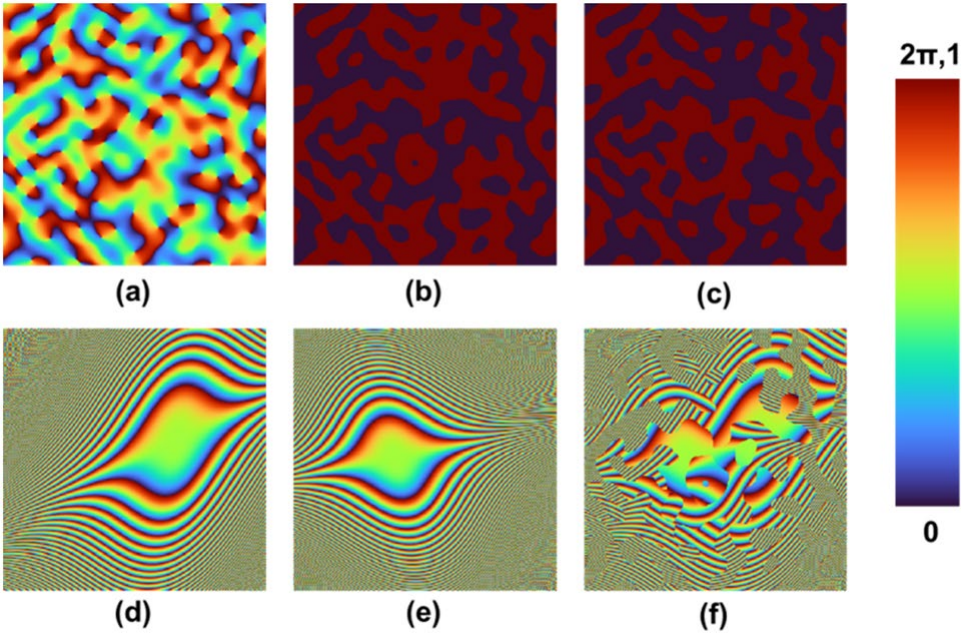
Design of the randomly multiplexed phase mask. (**a**) Random matrix *S* varying between 0 and 1. (**b**) Binary random matrix *S*_1_ and (**c**) complementary binary random matrix *S*_2_. Phase distribution of phase modulators (**d**) PM_1_ and (**e**) PM_2_. (**f**) Random phase multiplexed mask *S*_1_ × PM_1_ + *S*_2_ × PM_2_. The images are generated using MATLAB (version R2020b)^36^.

In the previous section, the PM is considered a pure phase function, but realizing a phase PM that projects several Airy beams is not trivial. In our previous studies on I-COACH, the PM for creating controlled scattering and random arrays of dots was designed using the Gerchberg-Saxton algorithm (GSA)^16,17,25,38^. In this study, a random phase multiplexing method was adapted from^5^. The phase distribution of the PM is designed as ${\Phi }_{OM}=\sum_{k=1}^{N}{S}_{k}exp\left[-j\frac{2\pi }{\lambda }\left\{{\xi }_{k}{\left({x}_{k}+\Delta {x}_{k}\right)}^{3}+{\eta }_{k}{\left({y}_{k}+\Delta {y}_{k}\right)}^{3}\right\}\right],$ where ${x}_{k}={x}_{0}cos{\theta }_{k}+{y}_{0}sin{\theta }_{k}$, ${y}_{k}={y}_{0}cos{\theta }_{k}-{x}_{0}sin{\theta }_{k}$, $\Delta {x}_{k}$, and $\Delta {y}_{k}$ are the shifts, ${\xi }_{k}$, and ${\eta }_{k}$ are the scaling factors along the *x* and *y* directions, respectively, and *S* is the binary random matrix with values of 0 or 1 with a condition $\sum_{k=1}^{N}{S}_{k}=[1]$, where [1] is the matrix with identical values of 1. The design of the PM is pictorially represented for *N* = 2 in Fig. 2. The image of the random matrix $S\in \left[\mathrm{0,1}\right]$ and the two binary complementary random matrices *S*_1_ and *S*_2_ generated by selecting 1 for values above 0.5 and 0 otherwise are shown in Fig. 2a–c, respectively. The phase images of the two PMs, PM_1_ and PM_2_, for generating two different Airy beams are shown in Fig. 2d,e, respectively. The randomly multiplexed phase mask given as *S*_1_ × PM_1_ + *S*_2_ × PM_2_ is shown in Fig. 2f, where ‘× ’ refers to element-wise multiplication. In this way, it is possible to create a phase-only modulator with easier implementation in comparison to a complex modulator^28^. A relatively narrow-band random matrix has been used to demonstrate the concept in Fig. 2. In the following experiment, the random matrix has a wider bandwidth dictated by the pixel size of the dynamic device used for random multiplexing.

**Figure 3:**
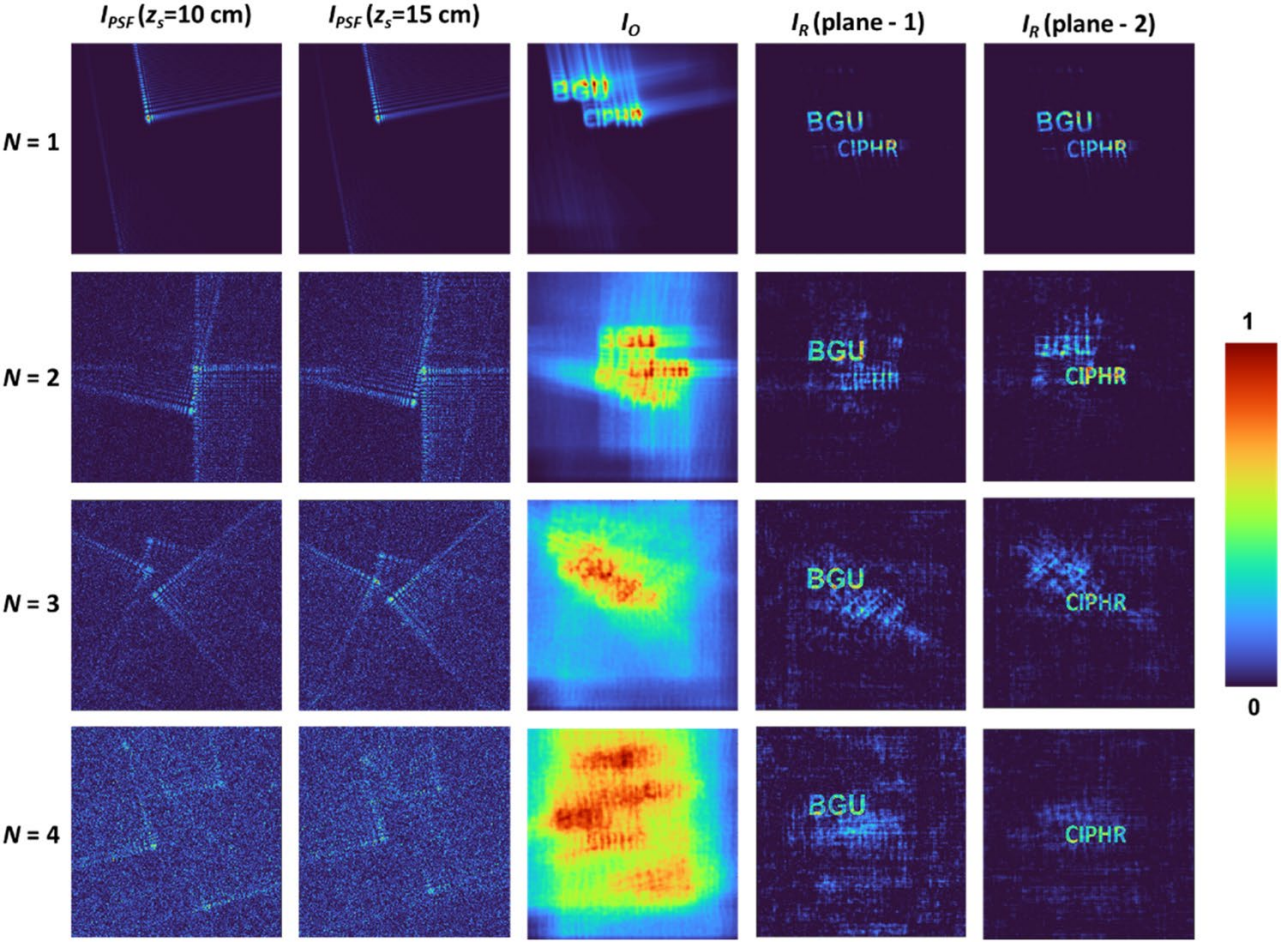
3D simulation results. Simulation results for two test objects located at two axially separated planes using a different number of Airy beams. *z*_*s*_ is the distance between the object and the PM; *N*—number of Airy beams; *I*_*PSF*_—point spread function; *I*_*O*_—object intensity distribution; *I*_*R*_—reconstructed images. Numerical simulations are performed using MATLAB (version R2020b)^36^.

A simulation study is carried out next with a matrix size of 500 pixels along the *x* and *y* directions, pixel size of 10 µm, *λ* = 650 nm, *z*_*s*_ = 10 cm, *z*_*h*_ = 10 cm and PM diameter of *D* = 5 mm. The shifting and scaling factors of the Airy beams were varied randomly with respect to one another to create a random set of Airy beams. Only the cases where the Airy beam patterns were present within the sensor area were selected. Two test objects, namely, ‘BGU’ and ‘CIPHR’, were mounted at two different planes separated by a distance of 5 cm. The indirect imaging process with Airy beams with *N* = 1 to 5 and NLR has been investigated. The images of the *I*_*PSF*_ at two planes, the object intensity distribution, and the reconstruction results corresponding to the two planes using NLR for *α* = 0 and *β* = 0.6 are shown in Fig. 3. According to the rightmost columns of Fig. 3, the axial resolution improves with an increase in the number of beams, as also demonstrated in^27^. It can also be observed that the lateral resolution does not vary with changes in axial resolution, but the background noise seems to increase with the number of Airy beams. Cases *N* = 1 and *N* = 2 have a low axial resolution, so in this study, cases *N* = 3 and *N* = 4 are investigated. The variation in axial resolution with the number of beams was more gradual in the case of Bessel beams^28^ than in the case of Airy beams, which can be attributed to the non-linear optical paths of the Airy beams. When the 3D location of a point changes, the Airy beams drift relatively in a random direction from one another, resulting in a rapid change in axial resolution with the number of beams.

## Experiments

**Figure 4:**
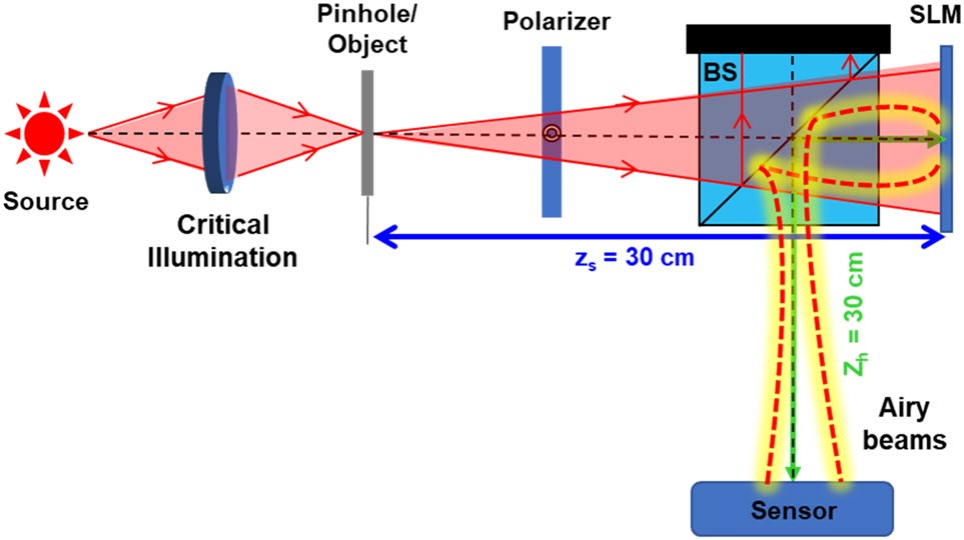
Schematic of the experimental setup. An object is critically illuminated using an incoherent source, and the light from the object is incident on an SLM through a BS. The phase mask displayed on the SLM converts the incident light into a sparse random array of Airy beams, which is captured by an image sensor. BS—beam splitter; SLM—spatial light modulator; *z*_*s*_—distance between object and SLM; *z*_*h*_—distance between SLM and sensor.

The schematic of the experimental setup is shown in Fig. 4. An incoherent source (Thorlabs LED625L, 12 mW, λ = 635 nm, ∆λ = 15 nm) was used to critically illuminate the object, elements 5 and 6 (‘digits’ and ‘gratings’ both) of group 2 of the negative USAF target. The 3D experiments were performed on the configuration of a single channel by combining the two objects with an axial spacing of 5 cm between them. A phase-only spatial light modulator (SLM) (Holoeye PLUTO, 1920 × 1080 pixels, 8 µm pixel pitch, phase-only modulation) was used to modulate the light beam by displaying the PM. A polarizer was used to allow light only along the active axis of the SLM. The intensity images after modulation are captured by a digital camera (Retiga R6-DCC3260M, pixel size 4.54 × 4.54 μm, exposure time 30 ms) placed 30 cm from the SLM. A pinhole with a size of 15 µm was used to record the PSF library. The USAF objects were then mounted at 30 cm and 25 cm in front of the SLM, and the intensity distribution was recorded by the image sensor only once.

**Figure 5:**
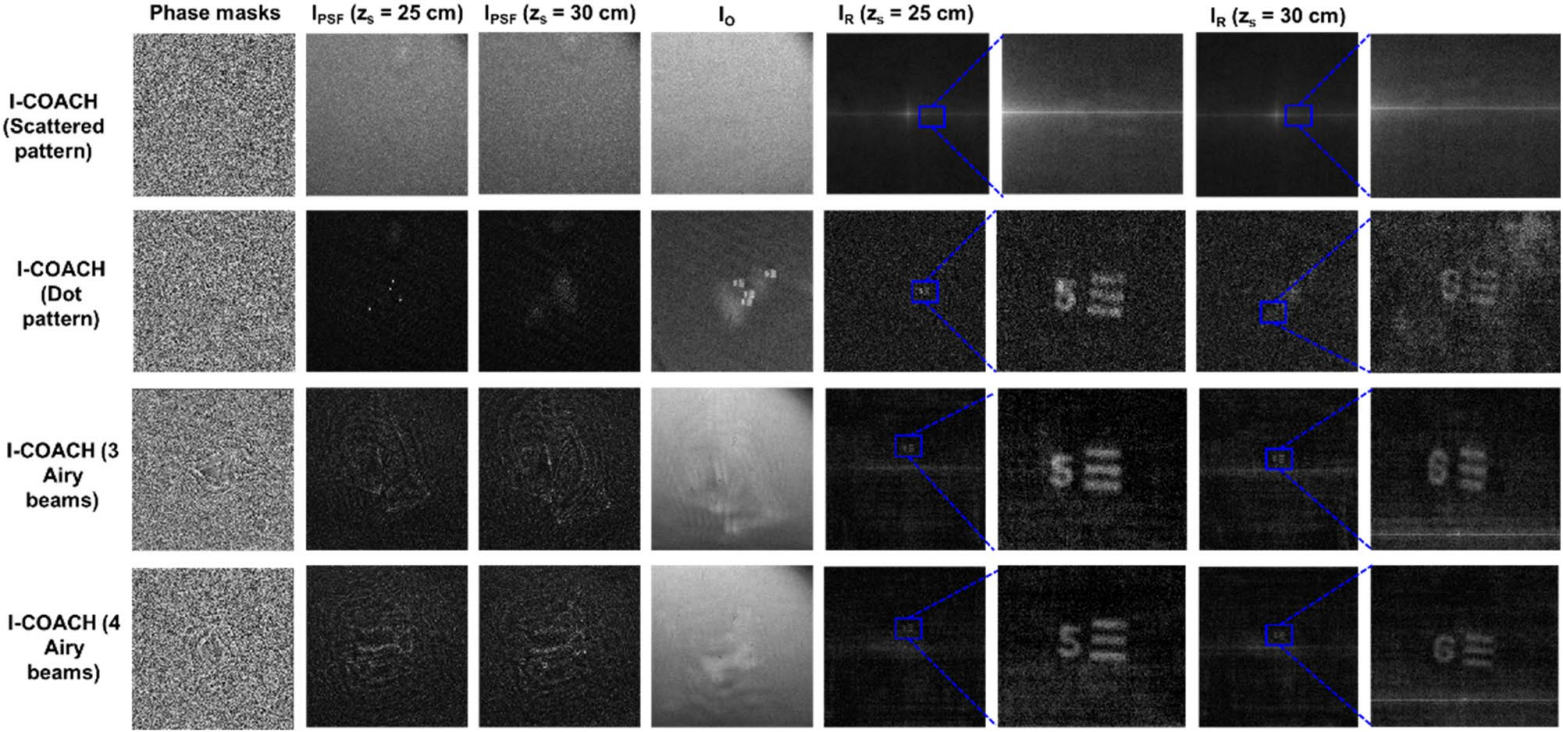
Experimental results of I-COACH with scattered pattern, dot pattern, 3 Airy beams and 4 Airy beams. *z*_*s*_ is the distance between the object and the phase modulator; *I*_*PSF*_—point spread function; *I*_*O*_—object intensity distribution; *I*_*R*_—reconstructed images.

For a reliable study, the proposed method was compared with the original I-COACH with a scattered pattern^16^ and with a random dot pattern^25^. In the previous demonstrations of I-COACH with a scattered pattern, multiple recordings with independent masks were carried out to reduce the background noise in addition to the pure phase condition applied in the design of the phase mask using GSA. In this study, only the number of Airy beams has been changed, and their locations have not been optimized to achieve a high SNR. Therefore, I-COACH with a scattered pattern and a dot pattern has been used as raw as possible without any pre- and post-processing. The 3D imaging results of I-COACH with 3 and 4 Airy beams were compared with I-COACH with a scattered pattern and a dot pattern, as shown in Fig. 5. I-COACH with a random phase mask has a higher background noise in comparison to I-COACH with a dot pattern and Airy beams. Furthermore, the results of 3 and 4 Airy beams were found to be better than I-COACH with a dot pattern. The line-like intensity patterns seen in the reconstruction results are the reconstruction noises of non-linear reconstruction.

## Conclusion

In summary, we have developed a 3D single-shot incoherent imaging technique using a coded phase mask that can map an object point into an array of Airy beams. With an increase in the number of Airy beams, the axial resolution is improved, while the lateral resolution remains constant, offering independence between the two types of resolutions, which does not exist in many incoherent holography systems. The technique has been compared with the previous versions of I-COACH with a scattered pattern and dot pattern, and the proposed method was found to perform better in terms of the signal-to-noise ratio. It is possible to improve the performance of I-COACH with a dot pattern by multiplexing multiple mask patterns with different focal lengths, but the above procedure cannot enable 3D imaging continuously over an entire depth with the same lateral resolution^39,40^. The computational reconstruction of object images can be carried out using different types of filters and algorithms such as matched filters, phase-only filters, Weiner filters, the Lucy-Richardson algorithm, and the recently developed Lucy-Richardson-Rosen (LRRA) algorithm^41^. It has already been well-established that NLR performs better than the rest of the methods for scattered intensity distribution, except for simple spatially symmetric PSFs, such as in the cases of synchrotron imaging^41^ and lens-based imaging^42^. In this study, NLR was found to perform better than LRRA, as the PSF is not spatially symmetric. We believe that the proposed technique will find applications in fluorescence microscopy, astronomical imaging, and holography. Lensless imaging systems comprising only a thin mask plate attached closely to an image sensor such as FlatCam^43^ and Fresnel zone aperture lensless imagers^44^ cannot perform 3D imaging. However, revised studies of these methods using the proposed approach might be realized in the future.

## Data Availability

The datasets used and/or analysed during the current study are available from the corresponding author on reasonable request.
